# Atomic Defect Induced Saturable Absorption of Hexagonal Boron Nitride in Near Infrared Band for Ultrafast Lasing Applications

**DOI:** 10.3390/nano11123203

**Published:** 2021-11-26

**Authors:** Chen Cheng, Ziqi Li, Ningning Dong, Rang Li, Jun Wang, Feng Chen

**Affiliations:** 1 Shandong Provincial Key Laboratory of Optics and Photonic Devices, School of Physics and Electronics, Shandong Normal University, Jinan 250014, China; 2 State Key Laboratory of Crystal Materials, School of Physics, Shandong University, Jinan 250100, China; drziqili@163.com (Z.L.); sdurangli@163.com (R.L.); 3 Key Laboratory of Materials for High-Power Laser, Shanghai Institute of Optics and Fine Mechanics, Chinese Academy of Sciences, Shanghai 201800, China; n.n.dong@siom.ac.cn (N.D.); jwang@siom.ac.cn (J.W.)

**Keywords:** two-dimensional materials, hexagonal boron nitride, atom-scale defect, saturable absorbers, mode-locked lasers

## Abstract

Defect-induced phenomena in 2D materials has received increasing interest among researchers due to the novel properties correlated with precise modification of materials. We performed a study of the nonlinear saturable absorption of the boron-atom-vacancy defective hexagonal boron nitride (h-BN) thin film at a wavelength of ~1 μm and its applications in ultrafast laser generation. The h-BN is with wide band gap of ~6 eV. Our investigation shows that the defective h-BN has a wide absorption band from visible to near infrared regimes. First-principle calculations based on density functional theory (DFT) indicate that optical property changes may be attributed to the boron-vacancy-related defects. The photoluminescence spectrum shows a strong emission peak at ~1.79 eV. The ultrafast Z-scan measurement shows saturable absorbance response has been detected for the defective h-BN with saturation intensity of ~1.03 GW/cm^2^ and modulation depth of 1.1%. In addition, the defective h-BN has been applied as a new saturable absorber (SA) to generate laser pulses through the passively Q-switched mode-locking configuration. Based on a Nd:YAG waveguide platform, 8.7 GHz repetition rate and 55 ps pulse duration of the waveguide laser have been achieved. Our results suggest potential applications of defective h-BN for ultrafast lasing and integrated photonics.

## 1. Introduction

Artificial atomic defects in materials provide a powerful approach to establish physical systems for quantum technologies, including quantum communications, computing, information processing, etc. [1,2,3,4]. Up to now, they are widely applied in a few wide-bandgap (WBG) semiconductors such as diamond, ZnS, and silicon carbide [5,6,7,8,9,10]. With extended research of graphene, two-dimensional (2D) materials of single-atom-layer scale—such as transition metal dichalcogenides (TMDCs) or diselenides—have drawn great attention due to their layer-dependent properties for applications in various electronic and photonic devices [11,12]. Similarly to graphene, hexagonal boron nitride (h-BN) has a hexagonal honeycomb structure compound of boron and nitrogen atoms. Instead of the ultrahigh electrical conductivity of graphene, h-BN applies as an insulator with a wide bandgap of ~6 eV, which is of significant difference with the zero-direct-bandgap graphene [13,14,15,16,17,18,19,20,21]. The h-BN can be a complementary substrate to graphene and other 2D materials, which can effectively reduce the lattice mismatch and improve the uniformity of 2D materials such as graphene, and can also be used as an isolator at visible (VIS) and infrared (IR) wavelengths [16,17,22,23,24,25,26]. In addition, inducing defects in h-BN may overcome its shortcomings and enrich its applications in UV-VIS as well extend to longer-wavelength ranges [27,28,29,30]. Like other 2D materials, h-BN possesses fascinating photoluminescence (PL) and nonlinear optical (NLO) properties [14,31,32,33]. However, in defective h-BN, such properties correlated to the defects have not been fully explored yet. One of the most interesting NLO features of 2D atom-scale materials is the nonlinear saturable absorption, which is essential for the generation of ultrafast laser pulses through the passive Q-switching or mode-locking process [34,35,36]. The 2D-materials-based pulsed laser systems have been widely implemented in the laser platforms of bulks, fibers, and waveguides, in which a number of 2D materials—including graphene, TMDCs, topological insulators, black phosphorus, etc.—have been applied as efficient saturable absorbers (SA) [37,38,39,40,41,42,43]. As for h-BN, the wide bandgap of ~6 eV restricts its applications to a certain extent in long-wavelength regimes. Kumbhakar et al. reported UV-VIS NLO property of h-BN nanosheets and mentioned its possible saturable absorption in VIS band [27]. Kislyakov et al. reported two-photon absorption of h-BN in near infrared (NIR) [44]. Extension of applications to low-energy photon regimes can be realized by the introduction of defects in wide bandgap materials [29,45,46,47]. In fact, defect-engineered 2D layered materials have been applied in versatile areas with modified, or sometimes enhanced, properties in comparison to their perfect counterparts [48,49,50,51]. Cai et al. reported h-BN as SA material in a passively Q-switched erbium-doped fiber laser operating at 1.5 µm, in which they changed the ratio of boron atom and nitrogen atom [52]. In this work, we investigated the NLO properties of a defective h-BN in NIR wavelength band. The nonlinear saturable absorption of h-BN at near infrared wavelength (1 μm) has been observed, which is of potential applications for the NIR lasing. The mechanisms of such nonlinear saturable absorption in defective h-BN have been explored, which may be attributed to the boron-atom vacancy (Bv). We also further apply defective h-BN as a SA to generate ultrafast lasers at a 1 μm wavelength. The results herald that the applied range of Bv-defective h-BN can extend up to 2 μm; and Bv-defective h-BN may have a great potential to achieve on-chip integrated devices of ultrafast photonics

## 2. Materials and Methods

**Figure 1:**
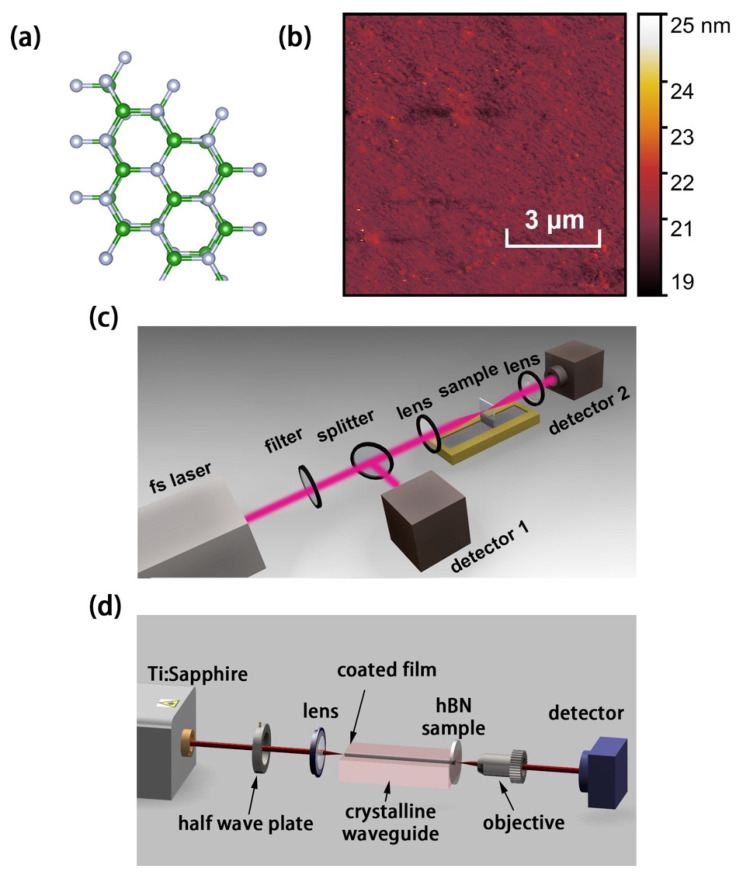
(**a**) Typical defect-free h-BN atomic structure; (**b**) AFM image of h-BN sample; the schematic of the experimental setup of (**c**) the Z-scan system and (**d**) the waveguide laser platform in this work.

The defective h-BN thin film used in this work was customized from a chemical vendor (6Carbon Technology Co., Ltd., Shenzhen, China). It was made by chemical vapor deposition (CVD) technology, coated on a 10 × 10 mm^2^ surface of a sapphire wafer of which the faces were optically polished. The h-BN thin film was ensured to be consecutive and to cover all the face completely. Figure 1a shows a typical free-defect h-BN atomic structure that is very similar to hexagonal structure of graphene. An atomic force microscope (AFM) was utilized to characterize the defective h-BN thin film (shown in Figure 1b), by which it was observed that the thin film was with few wrinkles. Via AFM measurement, we obtained the height between the sapphire substrate and the sample surface, the value was approximately 13 nm. To investigate the crystalline structure, a home-made Raman spectral microscope was used to inspect the Raman scattering signal at 532 nm laser exciting. An optical microscope (Olympus BX43) connected with a fiber spectrometer (NOVA, Ideaoptics, Co., Ltd., Shanghai, China, 8 cm^−1^ resolution, 175 to 3100 cm^−1^) was employed to obtain the Raman spectrum of h-BN sample. A set of filters (cut-off at 186 cm^−1^, OD > 6 at 532 nm) was used to block the excitation light signal and to distinguish the scattered Raman signal. The detected region from 1000 cm^−1^ to 3000 cm^−1^ was set for measurement, including first- and second-order Raman-active spectra of the h-BN sample. As well as utilizing this microscope system, photoluminescence (PL) of the sample can be carried out via changing detective spectrometers (NOVA-EX, Ideaoptics, Co., Ltd., Shanghai, China, 1.98 μm resolution, 325 to 1100 nm) and filters. The 532-nm-wavelength laser was focused to be a spot with an approximate 1.5-μm diameter on the sample surface by an objective (100×, 0.8 N.A.). Additionally, this objective was used to collect Raman and PL signals into a 100-μm-diameter fiber. The h-BN sample was placed on a three-axis motorized stage (0.2-nm-resolution) of the microscope system.

For further investigation on the linear absorption, an absorption spectrum of the h-BN sample was measured by using a UV/VIS/NIR spectrophotometer (UV1800, Shimadzu, Kyoto, Japan) from 200 nm to 1100 nm, with a resolution of 1 nm. By this spectrophotometer, we performed measurement of not only the absorption, but also the transmittance and reflectance as functions of incidence wavelength.

For studying the nonlinear absorption of the h-BN sample, an open-aperture Z-scan system was used, that depicted in Figure 1c. In this system, the sample was mounted on a motorized translation stage to traverse the incident beam. The beam was focused by a 150 mm-focal-length lens. It was gradually moving, passing along the *z*-axis (laser propagation direction) for obtained the transmittance as a function of the incident laser intensity through the focus.

A waveguide laser platform was employed to study the saturable absorption of thin film as a SA in waveguide lasing, shown in Figure 1d. The platform is built by a Nd:YAG crystalline waveguide (doped by 1 at % Nd^3+^ ions), fabricated by the femtosecond laser writing and coated optical film [39,53]. A 30 mm-focal-length lens was used to launch the pump beam. Additionally, a 20× and 0.4-N.A. microscope objective was used to collect the output lasers. A tunable CW Ti:Sapphire laser (Coherent MBR PE) generates optical pump beam with a linearly polarized light beam at 810 nm wavelength. The h-BN sample is covered on an output coupler mirror. The mirror and h-BN sample were pressed close to the end-face of the waveguide. The detective devices were located after the objective lens at the end of the waveguide lasing system, including a fast photodiode, a power meter, a spectrometer, etc.

## 3. Results and Discussion

**Figure 2:**
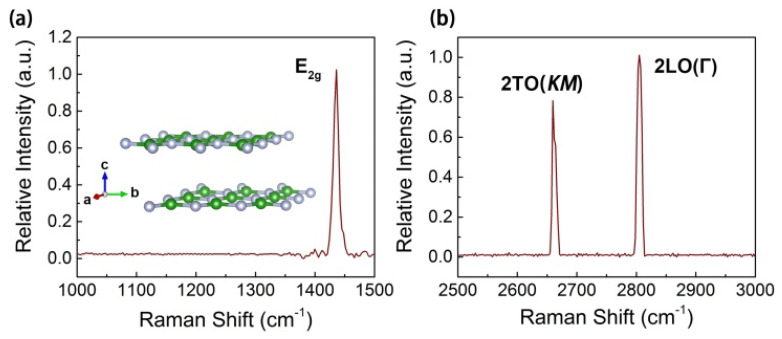
(**a**) First-order Raman spectrum, the inset shows crystalline structure of two layer h-BN prototype; (**b**) second-order Raman spectrum.

Figure 2a shows the first-order Raman spectrum of h-BN sample excited with 532 nm. The inset displays the crystalline structure of bi-layer h-BN prototype. The atomic displacements of boron and the nitrogen atoms in each plane move in opposite directions symmetrically that give to a Raman-active peak, as well without LO-TO splitting since the contributions from the two planes cancel each other [54,55]. Accordingly, this typical h-BN high-energy peak appeared at 1435.4 cm^−1^. This peak demonstrated an in-plane optical mode with the E_2g_ or Г symmetry, which differed from two peaks of c-BN. The second-order Raman spectrum has been shown in Figure 2b, in the range from 2500 cm^−1^ to 3000 cm^−1^. Two peaks have appeared at 2661 cm^−1^ and 2805.1 cm^−1^, which corresponded to 2TO mode and were twice the frequency of the Raman-active Г point mode. These three peaks are consistent with the previous report and characterized a typical h-BN sample [54,56].

**Figure 3:**
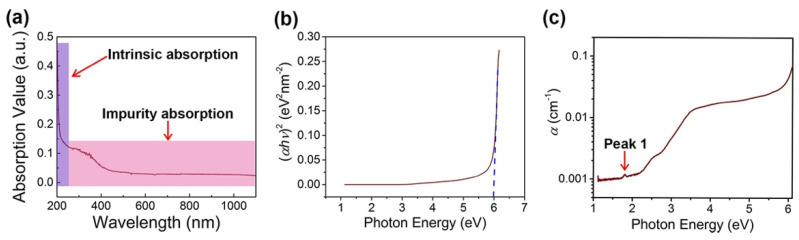
(**a**) Linear absorbed spectrum with the wavelength from 200 nm to 1100 nm; (**b**) (*αhν*)^2^-*hν* spectrum of the h-BN sample based on Tauc’s plot; (**c**) calculated linear absorbed coefficient α as a function of photon energy.

A linear absorbed spectrum with the wavelength from 200 nm to 1100 nm is shown in Figure 3a. In this spectrum, it can obviously find that the intense impurity absorption appeared from the VIS band to NIR band. Figure 3b shows the (*αhν*)2-*hν* spectrum of the h-BN sample based on Tauc’s plot [57]. By drawing a tangent line in the linear region of the spectrum, the line is extrapolated to *hν*-axis. The intercept of ~6 eV was obtained for this sample, that the value would be the direct band-gap of the h-BN sample. The result is in agreement with the previous works [58]. The linear absorbed coefficient *α* has a relationship with the transmittance *T* and thickness *d* of the sample, which can be expressed as

(1) α=ln(1/T)/d

The calculated linear absorbed coefficient *α* is a function of photon energy, which has been shown in Figure 3c. In this spectrum, there are a few absorbed impurity energy levels appearing at from VIS to NIR regions, in which a peak is marked in the figure as Peak 1 at 1.8 eV.

**Figure 4:**
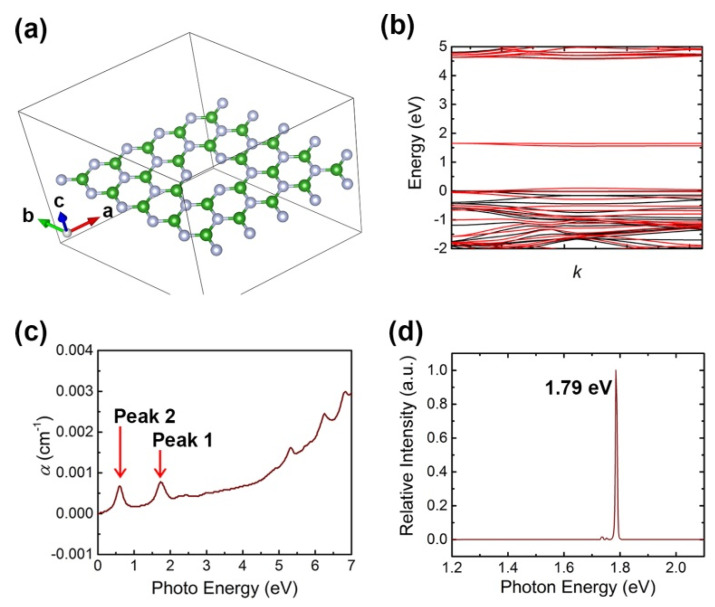
(**a**) Bv-defective h-BN structure of a unit cell; (**b**) spin-polarized band structures of calculated h-BN with single B-vacancy; (**c**) calculated spectrum of a function between photo energy and linear absorption coefficient *α*; (**d**) PL spectrum excited with 532 nm.

To reveal the origin of impurity absorption, we use first-principle calculations based on density function theory (DFT) to investigate the defects using the Vienna ab initio simulation package (VASP) with the Perdew–Burke–Ernzerhof (PBE) exchange-correlation functional. The plane wave was set to the cut-off energy of 500 eV. All atoms were allowed to relax until the Hellmann–Feynman forces reached the convergence criterion of less than 0.01 eV/Å. The convergence threshold of energy was set at 10^−5^ eV. The Monkhorst-Pack scheme was used to sample k-points in the Brillouin zone. After a few calculations of possible defective structures, a Bv defect was considered in the h-BN sample, which played a main part in the impurity absorption. Figure 4a shows the Bv-defective h-BN structure of a unit cell and the detailed defective structures are shown in Figure S1 and Figure S2 (Supplementary Materials). The spin-polarized band structures of BN with single B-vacancy are shown in Figure 4b, in which the Fermi level has been set to zero. Spin-up and spin-down structures are represented by red and black lines. According to projector-augmented wave (PAW) method that the light is vertical respect to the plane of BN, and then the imaginary part *ε*_2_ of dielectric is determined by summation of all possible hopping processes [59]

(2) $$\varepsilon_{2}=\frac{4\pi^{2}e^{2}}{S}\sum_{c,v,k}2\omega \delta \left((\epsilon_{ck}-\epsilon_{vk})-\omega \right)\langle u_{ck}|u_{vk}\rangle \langle u_{vk}|u_{ck}\rangle$$

and the real part *ε*_1_ can be deduced by usual Kramers–Kronig transformation

(3) $$\varepsilon_{1}=1+\frac{2}{\pi }P\int_{0}^{\infty }\frac{\varepsilon_{2}(\omega^{\prime })\omega^{\prime }d\omega^{\prime }}{{\omega^{\prime }}^{2}-\omega^{2}+i\eta }$$

with these two, the absorption coefficients are given by

(4) $$I=\sqrt{2}\omega \sqrt{\sqrt{\varepsilon_{1}^{2}+\varepsilon_{2}^{2}}-\varepsilon_{1}}$$

Finally, we obtain a calculated spectrum of a function between photo energy and linear absorption coefficient *α*, which was shown in Figure 4c, the absorptions of other defective structures are shown in Figure S3 (Supplementary Materials). In this spectrum, two absorption peaks at ~1.8 eV and ~0.6 eV were labeled. Figure 4d shows PL spectrum excited with 532 nm. In this condition, a bright emission peak is detected at 1.79 eV (624 nm).

**Figure 5:**
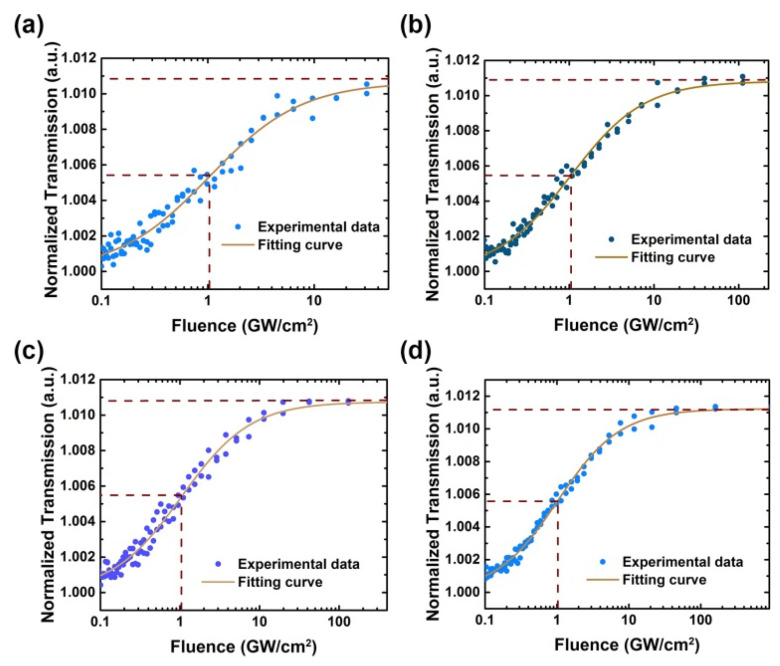
Nonlinear transmittance under incident (**a**) 50 nJ, (**b**) 80 nJ, (**c**) 100 nJ, and (**d**) 150 nJ detected by the Z-scan system.

In nonlinear absorption regions, conspicuous saturable absorption can be detected under incident 30–150 nJ femtosecond laser pulse in the Z-scan system. The original Z-scan data are shown in the Figure S4 (Supplementary Materials). The results were recalculated to be a function between incident fluence and transmission and are demonstrated in Figure 5a–d. The maximum incident energy was set to be 150 nJ, which was corresponding up to 160.3 GW/cm^2^ (*z* = 0). Despite being under such higher incident fluence, the defective h-BN exhibits a stable nonlinear performance, even though we repeated the Z-scan experiment continuously more than 10 times.

Based on the propagation theory, a differential equation in the thin film can be written as [34]

(5) $$\frac{dI}{dz^{\prime }}=-\alpha (I)I$$

where *z*′ is the propagation distance in the samples, *I* is the input intensity.

For nonlinear saturable absorption response, we can also describe the total absorption coefficient *α*(*I*) in Equation (1), the saturable absorption model, with the form of [60]

(6) $$\alpha (I)=\frac{\alpha_{0}}{1+I/I_{S}}$$

where *α*_0_ is linear absorption coefficient, and *I*_s_ is the saturable intensity. Normally, the normalized transmittance in the Z-scan measurement is

(7) $$T=\left(1-\frac{\alpha_{0}}{1+I/I_{S}}\right)/(1-\alpha_{0}d)$$

From the fitting curves based on Equation (7), a perfect saturable absorption data fitting can be found; the saturable intensity and modulation depth (*α*_0_*d*) are about 1.03 ± 0.01 GW/cm^2^ and 1.10% at 1030 nm.

**Figure 6:**
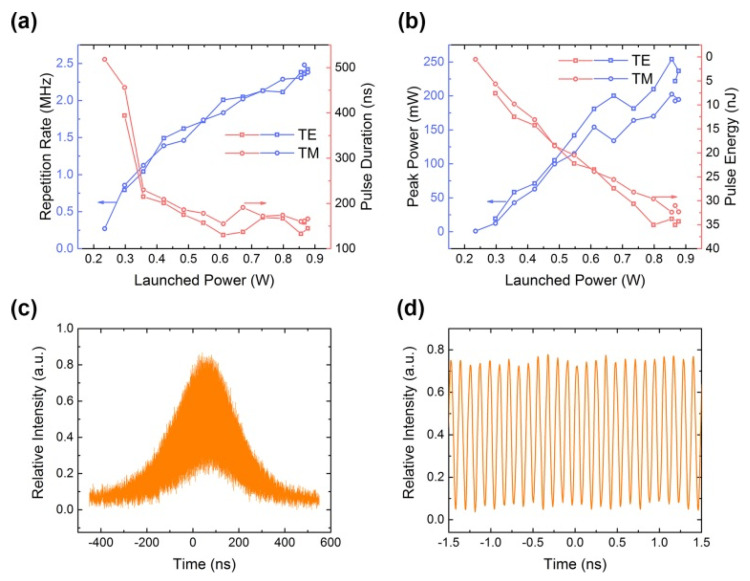
The waveguide laser parameters of Q-switched regimes, (**a**) repetition rates and pulse durations, (**b**) values of pulse energy and peak power, in TE- and TM-polarization respectively; the pulse trains output of (**c**) a Q-switched pulse envelope and (**d**) a mode-locking pulse train.

Using the waveguide lasing platform, a Q-switched pulsed laser was achieved under optical pump, with an SA of Bv-defective h-BN thin film. The working wavelength of waveguide lasing is 1064 nm (1.16 eV) for both TE- and TM-polarization. Figure 6a shows the repetition rates and pulse durations as a function of launched power at two orthogonal TE and TM polarizations. The maximum repetition rate values were 2.421 MHz and 2.481 MHz at TE- and TM-polarization, respectively. The minimum pulse duration values were as fast as 133 ns (158 ns) pumping at TE- (TM-) polarized laser. The pulse energy and peak power, as shown in Figure 6b, increased significantly as the incident pump power increased, from 0.5 nJ to 32.4 nJ (from 7.6 nJ to 35.0 nJ), in TE (TM) polarized light pumping, respectively. Figure 6b shows that the laser performance is unstable in areas where the pump power is higher than 0.85 W. High-power pump lasers may cause strong thermal effects, which may change the nonlinear performance of defective h-BN. The two-dimensional material SAs controlled by the defect state may have a broader application in integrated waveguide lasers and fiber lasers driven by lower optical power.

By further adjusting the resonant cavity of the waveguide lasing platform, a Q-switched mode-locked (QML) pulsed laser was obtained on the basis of the original Q-switched pulsed laser. The fast photodetector connected to the oscilloscope acquires the pulse signal of the mode-locked pulsed laser, as shown in Figure 6c,d. In Figure 6d, the mode-locked pulse has a repetition rate of 8.7 GHz and a pulse width of 55 ps. According to the selected linear Fabry–Pérot waveguide laser resonator, the repetition frequency of the laser oscillation in the cavity can be expressed as: *f* = *c*·2*nl*, where c (m·s^−1^) is the speed of light, n is the refractive index of the waveguide material, and l is the length of the cavity. The theoretical calculation repetition frequency f is ~8.7 GHz, which is consistent with the experimental results. Compared with graphene and other 2D materials SAs, the Bv-defective h-BN performs a fairly level lasing field. One percent modulation depth of Bv-detective h-BN is very similar to numerical values of most 2D materials, for example 1.1%- 1.8%-, and 0.8%-modulation-depth of graphene, WS_2_, and black phosphorous, respectively [61,62]. With pulse durations in picoseconds—for example 52 ps, 43 ps, 26 ps—CVD graphene, MoS_2_, and BiSe_2_, were obtained in QML regime [63]. In the results of DFT calculations, it can be speculated that the Bv-defective h-BN has a great potential in near 2 μm applications, which can be a complementary SA candidate of graphene SA [64].

**Figure 7:**
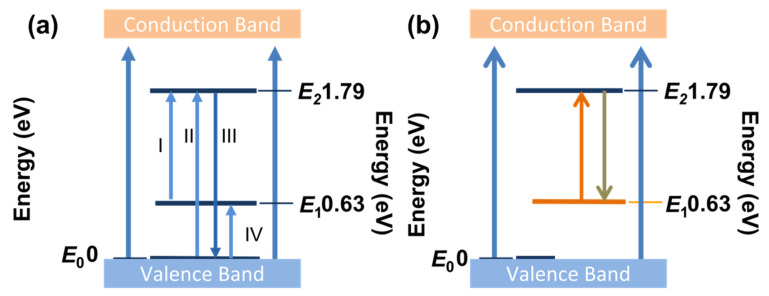
(**a**) Inferred the effective energy levels of Bv-detective h-BN; (**b**) a level system schematic of Bv-detective h-BN inducing saturable absorption.

Given the above results, it can be inferred that a series of Bv-detective energy levels are near the Fermi level of the h-BN sample, shown in Figure 7a. In this figure, four processes were labeled, which were E_1_ → E_2_, E_0_ → E_2_, E_2_ → E_0_, E_0_ → E_1_, respectively. According to the impurity absorption spectrum, E_2_ energy level is in the position of the impurity energy band. Process II and III have been described the impurity absorption process, which is corresponding to the cause of inducing Peak 1 in Figure 3c. The PL emission peak of 1.79 eV also can verify the existence of E_2_ energy level. The process III has been proved by calculation of Kramers–Kronig transformation, which also implied Bv-defective h-BN have applied potential in the NIR band of 2 μm. Based on the above processes, it may be inferred that a two-level system can be used to explain the saturable absorbed responses of defective h-BN, as shown in Figure 7b. In the figure, we labeled the process of absorption at working wavelength (1064 nm) in orange. In this system, when the h-BN sample was illuminated by the working laser, process IV occurred. When the intensity increased to the threshold of saturable absorption, excited state absorption would occur and induce the transition of E_1_ → E_2_ by a rapid depletion of the ground valence states. Accordingly, the number of electrons, which were in the ground state, approached zero. Then the h-BN sample no longer absorbed the working laser, so that it reached saturation and became transparent at the working wavelength.

## 4. Conclusions

To summarize, we have unveiled nonlinear saturable absorption from single-atomic defect of boron vacancy in two-dimensional h-BN. The single-atomic defect has caused a series of impurity absorption from VIS to NIR bands, as well induced a bright PL peak at 1.79 eV at room temperature. We used Z-scan technology to demonstrate the nonlinear saturable absorption of the defective h-BN at ~1 μm. Defective h-BN has a saturable intensity of 1.03 ± 0.01 GW/cm^2^ and a modulation depth of 1.10% at 1030 nm. Based on a Nd:YAG crystalline waveguide platform, the Q-switched mode-locking laser has been achieved with a 8.7 GHz repetition rate and 55 ps pulse duration. Our results improve the scientific and technological importance of artificial atom-defect in 2D materials, in particular h-BN. These will be a solid foundation for new ultrafast applications in photonic technologies and optoelectronics based on 2D materials, highlighting the great potential of h-BN devices.

## Data Availability

Data underlying the results presented in this paper are not publicly available at this time but may be obtained from the authors upon reasonable request.

## Supplementary Materials

The following are available online at https://www.mdpi.com/article/10.3390/nano11123203/s1, Figure S1: Chemical bond lengths (in Angstroms) in the Bv-defective hBN. The boron and nitrogen atoms are represented by green and grey balls respectively; Figure S2: The geometry structure of BN with (a) one N-atom vacancy, (b) B-atom vacancy and B_N_ substitution, (c) N-atom vacancy and N_B_ substitution. Red arrows mark the substitution sites; Figure S3: The calculated absorption coefficients of (a) Nv-defective, (b) NvB_N_-defective, and (c) BvN_B_-defective h-BN; Figure S4: Non-linear absorption responses detected by *Z*-scan system with (a) 50 μJ, (b) 80 μJ, (c) 100 μJ, and (d) 150 μJ incident intensities.
