# Supplementary material for: Atomic Defect Induced Saturable Absorption of Hexagonal Boron Nitride in Near Infrared Band for Ultrafast Lasing Applications
Source: Nanomaterials (Basel). 2021 Nov 26;11(12):3203. doi: 10.3390/nano11123203 (PMC8707294; doi:10.3390/nano11123203)
Supplement: Supplementary file 1 [file nanomaterials-11-03203-s001.zip › nanomaterials-1448884-supplementary.pdf]

nanomaterials

Article

Atomic Defect Induced Saturable Absorption of Hexagonal Boron Nitride in Near Infrared Band for Ultrafast Lasing Applications

Chen Cheng [1,*], Ziqi Li [2], Ningning Dong [3], Rang Li [2], Jun Wang [3] and Feng Chen [2,*]

1 Shandong Provincial Key Laboratory of Optics and Photonic Devices, School of Physics and Electronics, Shandong Normal University, Jinan 250014, China
2 State Key Laboratory of Crystal Materials, School of Physics, Shandong University, Jinan 250100, China; drziqili@163.com (Z.L.); sdurangli@163.com (R.L.)
3 Key Laboratory of Materials for High-Power Laser, Shanghai Institute of Optics and Fine Mechanics, Chinese Academy of Sciences, Shanghai 201800, China; n.n.dong@siom.ac.cn (N.D.); jwang@siom.ac.cn (J.W.)
* Correspondence: drccheng@sdnu.edu.cn (C.C.); drfchen@sdu.edu.cn (F.C.)

Calculations of Defective Absorption

Figure S1 shows the optimized structure for the boron-vacancy (Bv) defect in hexagonal boron nitride (h-BN), in which the bond lengths have been labelled.

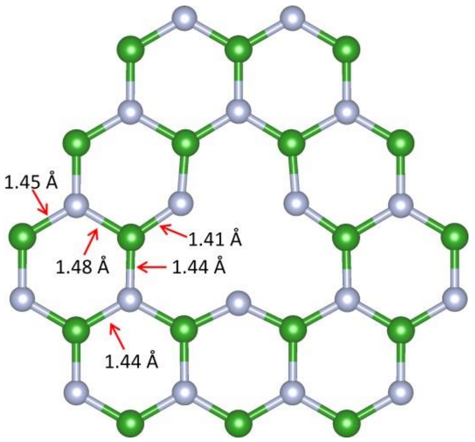

Figure S1. Chemical bond lengths (in Angstroms) in the Bv-defective hBN. The boron and nitrogen atoms are represented by green and grey balls respectively.

For investigating the defects in h-BN, we may consider the most likely candidates of defective structures, not only the Bv detect. Figure S2 a–c show other three defective structures including nitrogen-vacancy (Nv), an anti-site complex in which the boron atom substitutes the nitrogen atom and there is a missing atom at the boron site (NvB_N), and corresponding BvN_B (the nitrogen atom substitutes the boron atom).

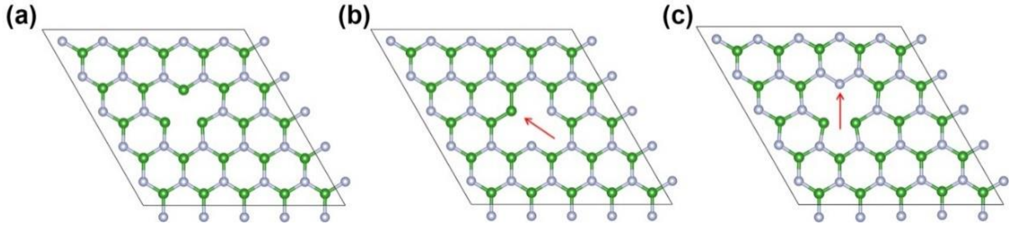

Figure S2. The geometry structure of BN with (**a**) one N-atom vacancy, (**b**)B-atom vacancy and B_N substitution, (**c**) N-atom vacancy and N_B substitution. Red arrows mark the substitution sites.

All possible defects have been calculated by Vienna ab initio simulation package (VASP) as same conditions in the main text. According to projector-augmented wave (PAW) method, the transmissions finally can be calculated. Figure S3 a,b show the transmission spectra under the conditions of spin-polarized solutions.

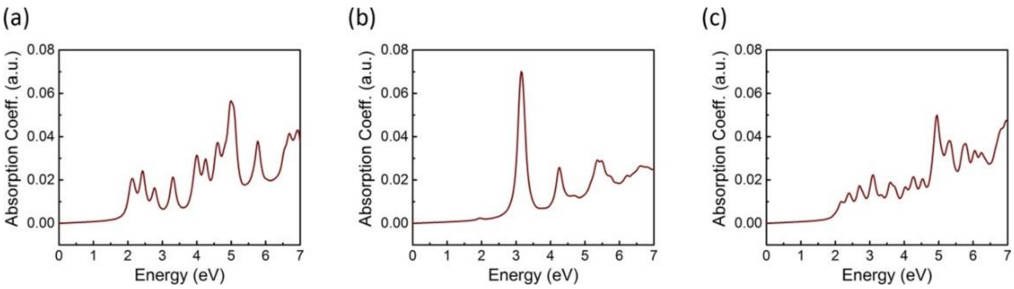

Figure S3. The calculated absorption coefficients of (**a**) Nv-defective, (**b**) NvB_N-defective and (**c**) BvN_B-defective h-BN.

Non-Linear Transmission Responses

Figure S4 a–d show non-linear transmission responses detected by the Z-scan system with 50 μJ, 80 μJ, 100 μJ and 150 μJ incident intensities, respectively.

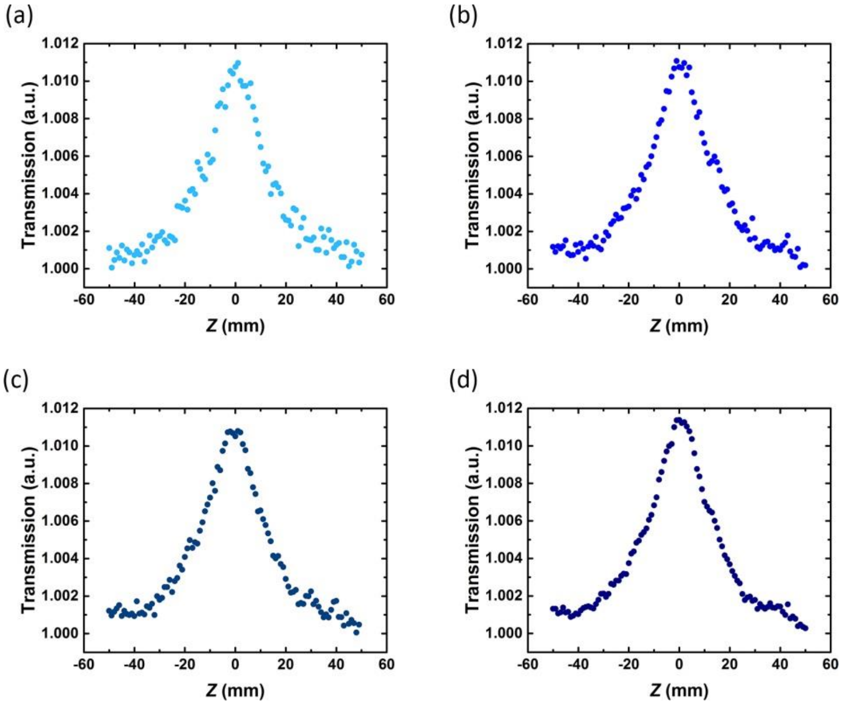

Figure S4. Non-linear absorption responses detected by *Z*-scan system with (**a**) 50 μJ, (**b**) 80 μJ, (**c**) 100 μJ, and (**d**) 150 μJ incident intensities.
